# A self-assembled two-dimensional hypersonic phononic insulator

**DOI:** 10.1515/nanoph-2025-0141

**Published:** 2025-10-15

**Authors:** Pedro Moronta, Sandeep Sathyan, Edson R. Cardozo de Oliveira, Rafael J. Jiménez-Riobóo, Norberto Daniel Lanzillotti-Kimura, Pedro D. García, Cefe López

**Affiliations:** Instituto de Ciencia de Materiales de Madrid (ICMM), Calle Sor Juana Inés de la Cruz 3, 28049 Madrid, Spain; Consejo Superior de Investigaciones Científicas (CSIC), Madrid, Spain; Escuela de Doctorado, Universidad Autónoma de Madrid, Madrid, Spain; Université Paris-Saclay, CNRS, Centre de Nanosciences et de Nanotechnologies, 10 Boulevard Thomas Gobert, 91120 Palaiseau, France

**Keywords:** optomechanics, phonon transport, ultra-fast pump and probe techniques, phonon insulator, self-assembly

## Abstract

The coupling between the electromagnetic field and the motional degrees of freedom of a nanoscale object offers an interesting control-knob for light–matter interaction. However, this interaction is rather weak so, in general, a precise control over the mechanical vibrations of the object becomes desirable for enhancing and fine-tuning this coupling. Phonon insulation is a base-line to achieve nanoscale vibration control by inducing constructive and destructive phonon interference, which opens phononic band gaps at various frequencies. Phonon insulators are essential for managing and directing mechanical vibrations in advanced mechanical systems, such as resonators and acousto-optic modulators. Conventional fabrication methods, such as electron-beam lithography, are highly robust and efficient but also very demanding and limited in accessibility due to their cost and technical requirements. Self-assembly offers an alternative route for fabricating mechanical structures from diverse materials, allowing for innovative designs with improved functionalities. By using natural self-organizing processes, production is simplified and accelerated, with components assembling automatically in a low-cost, scalable manner. Here, we present a simple self-assembled device that functions as a phonon insulator in the GHz frequency range and can be easily integrated onto silicon. This integration opens the door to a wide range of applications, including optical communication, quantum computing, and ultra-sensitive sensors, where efficient control over mechanical vibrations is vital.

## 1 Introduction

Phonons determine diverse physical phenomena such as thermal conductivity [1], mechanical stability [2], and acoustic properties [3], but also mediate the response of the material with external excitations, particularly with light [4]. While this coupling is challenging and weak, it also offers a control mechanism to interact with the material and to engineer novel material properties. To control the elastic coupling at the nanoscale, it is crucial to confine phonons efficiently within a small volume. A very efficient way to do this is by engineering gaps in nanostructures, a critical element achieved through precise mass distribution within the system. Phonon gaps effectively serve to inhibit phonon propagation and to enable phonon confinement and routing by cavities and waveguides [5].

Nanostructured materials are pivotal in enabling these elements. Their importance lies in their unique combination of selectively low mechanical coupling to the thermal environment and high spatial and spectral confinement of certain vibrations, making them particularly interesting for various applications such as those related to heat management [6] and hypersonic control [7]. A standard approach to achieve these characteristics is by the use of precise electron beam lithography of the materials, which require very fine control of the fabrication conditions in very clean environments, something not at hand easily. In addition, self-assembly is recognized as a manufacturing technique that streamlines and expedites production by allowing components to automatically assemble through self-organizing mechanisms [8]. This approach not only reduces costs but also significantly shortens production times, offering an efficient and accessible approach to nanoscale engineering. Over time, a variety of materials and shapes have been used with this technique, resulting in the creation of structures in one dimension, two dimensions, and three dimensions [9], [10], [11]. Researchers have discovered complete vibrational bandgaps characterized in 3D self-assembled structures [12], [13], [14], whereas 2D self-assembled structures have only demonstrated partial bandgaps limited to certain directions of propagation for which some frequencies are forbidden [15], [16].

Furthermore, this technique is potentially integrable with silicon photonics by the use of standard silicon wafers as the self-assembly substrate. This allows samples to undergo various manipulation processes, such as oxygen plasma or temperature treatments, without compromising the substrate’s properties. Additionally, silicon wafers are cost-effective and well-suited for large-scale manufacturing, making them both economical and practical for a wide range of applications. The merging of both domains, could enable the coupling of multi-scale optical resonators produced by different fabrication techniques to develop cost-effective devices boasting high-quality properties [17]. It has been shown that depositing self-assembled nanoparticles on thin substrates can mitigate energy losses into the substrate [18], while employing a thick substrate (100’s of nm thick) may increase them. However, the interaction of vibration modes from the monolayer and substrate, leads to the development of hybrid modes, thereby introducing additional bands among those of the monolayer [19]. By eliminating these collective modes, it becomes possible to observe a complete acoustic band gap.

In this study, we investigate the elastic modes of three distinct systems of self-assembled monolayers on a thick silicon substrate. The systems under study are: an arrangement of polystyrene spheres with a diameter of 202 nm; a modified sample in which the spheres have been reduced to 180 nm in diameter while maintaining the original 202 nm lattice constant; and a sample of 202 nm spheres that has been thermally treated to create prominent joints between neighboring spheres. These correspond, respectively, to the three configurations analyzed: touching, isolated, and overlapping spheres. We perform spectral and acoustic transport characterizations, complemented by computational analyses of the mechanical properties of the free-standing systems. Our results demonstrate that these free-standing systems can open a complete mechanical gap in the gigahertz (GHz) regime, with strong agreement observed between experimental measurements and computational predictions.

## 2 Results and discussion

**Figure 1:**
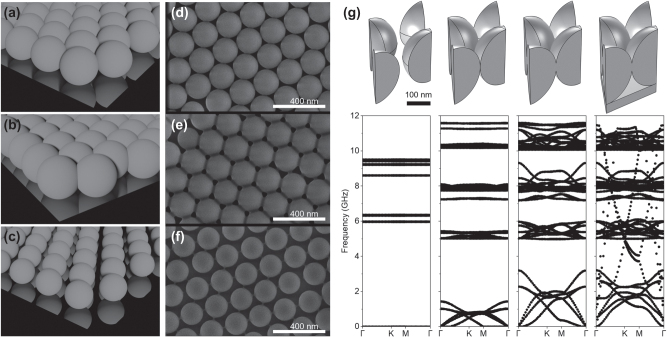
Schematic figure of the three monolayer configurations: (a) touching, (b) overlapping, and (c) isolated spheres. (d), (e) and (f) scanning electron microscope (SEM) images for the corresponding systems with 202 nm diameter polystyrene spheres. (g) Finite element numerical simulations showing the dispersion relation for the three different systems under study and the effect of overlapping spheres monolayer on top of a thin substrate.

To engineer the transport of hypersonic vibrations, we fabricate three kinds of samples. Figure 1(a)–(c) showcase the three systems analyzed here, each representing different arrangements of polystyrene microspheres, while maintaining the same lattice parameters for all three. The first system, 1(a), comprises an array of spheres in contact with each other, presenting limited but non-negligible mechanical interaction. The second system, 1(b), features an array of spheres partially overlapping with each other, forming a fully interconnected crystal structure in which the contact points are solid connections. The last system, 1(c), consists of an array of spheres that are entirely isolated from each other. To fabricate these three systems, we employ an air-liquid self-assembly technique applied to 202 nm diameter polystyrene micro-particles as the starting material [20]. After their self-assembly, two different processes lead separately to the desired isolated and overlapping spheres systems. Specifically, to achieve the isolated spheres, we subject a monolayer of spheres to oxygen plasma. This treatment, unlike reactive-ion etching [21], [22] or electron-beam lithography [23], effectively etches the polystyrene spheres isotropically [24], [25], subsequently reducing the spheres to approximately 180 nm. Conversely, the overlapping spheres are generated through a sintering process, involving controlled heating of the original monolayer at a controlled temperature and time. Additional details regarding the manufacturing process are available in the dedicated section. Scanning electron microscope (SEM) images of the samples are provided in Figure 1(d)–(f), showing the arrangement and morphology of the spheres within each system.

Figure 1(g) presents the calculated phononic dispersion relation of the structures, depicting the behavior of elastic waves in the system. This dispersion relation is obtained by solving the full three-dimensional elastic wave equation with finite element simulations conducted using commercial software. An additional panel is added to compare the vibrational dispersion relation of the systems studied here with one including a thin substrate, i.e., a substrate with a thickness comparable to the radius of the spheres. In the case of isolated spheres, the observed frequencies represent the resonant modes of individual spheres [26], [27], since the spheres are disconnected there is no dispersion and the bands appear flat. There is no phonon dispersion for this configuration. These frequencies can be finely tuned by adjusting parameters such as the diameter of the spheres and the material composition which varies the Young’s modulus [28]. The Young’s modulus of polystyrene exhibits significant variation among values reported in the literature, and it can also depend on the sphere diameter [29]. In our computational study, we determined the Young’s modulus by fitting the calculated dispersion bands of the isolated spheres to those measured experimentally using Brillouin spectroscopy. This was possible because the sphere diameter was accurately determined from SEM images. The Young’s modulus estimated through this fitting procedure was subsequently used consistently across all modeled systems in our computational study. The material parameters used in the simulations were: Young’s modulus *E* = 5.2 GPa, Poisson’s ratio *ν* = 0.34 and density *ρ* = 1,200 kg/m^3^. In the touching sphere system, where spheres are in partial contact, a nuanced interplay emerges. Here, spheres stick to one another, so that a continuous, structured medium is created whose periodicity breaks the elastic waves dispersion relation into bands and gaps. At high frequencies and despite this interaction, the individual vibrational eigenmodes of the spheres largely remain undisturbed. However, more significant differences emerge at low frequencies, where distinct bands arise corresponding to collective vibrational modes. In contrast, in the fully interconnected crystal structure, where the spheres overlap, the strong attachment between spheres significantly leads to wave propagation, even at high energies but particularly at lower frequencies. As the spheres form a continuous lattice, coupling and the hybridization of modes becomes more efficient. However, at higher frequencies, broader bands emerge due to increased complexity in the wave interactions within the crystal. The degree of overlapping between spheres determines the size of the band gap, providing an easily tunable pathway for adjusting the forbidden-frequency ranges. Finally, when the monolayer is lying on top of a thin substrate with a thickness comparable to the radius of the spheres, the complete mechanical gap is frustrated. This is evidenced by the emergence of two bands across the gap, as illustrated in the last panel of Figure 1(g). These phenomena highlight the complex relationship between the system’s structure and its mechanical properties, which determine the wave propagation in the final hybrid system. Vibrational modes of the membrane are influenced by adhesion to the monolayer, resulting in the formation of these hybrid modes as depicted in Figure S1 in the Supplementary Information. Hence, when manufacturing such structures, it is crucial to consider the final application. This entails determining whether a complete gap is needed or if reducing losses, even at the expense of sacrificing the other property, is preferable.

**Figure 2:**
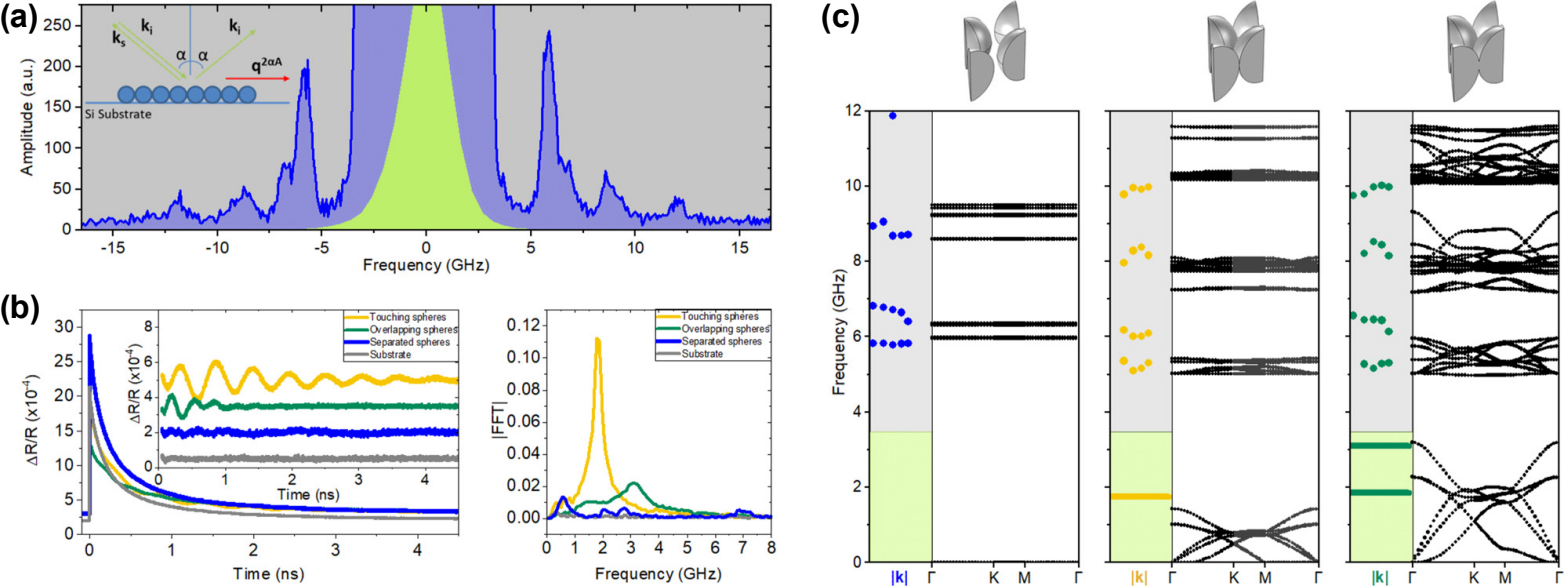
High-frequency modes are unveiled through Brillouin scattering measurements. (a) Typical Brillouin spectra for a monolayer of isolated polystyrene spheres with a diameter of 180 nm and a lattice constant of 202 nm. (b) Pump-probe measurements for the three systems and the silicon substrate. Fast Fourier transform (FFT) is applied to the experimental data to reveal the frequencies. (c) Finite element calculations are depicted by black circles, while experimental Brillouin measurements are illustrated with dots and pump-probe frequencies are plotted with thick color lines. Each dot from HRBS measurements corresponds to a different angle of incidence measurement hence scattering wavevector, while pump-probe data comes from normal incidence measurements tested for different positions within the sample.

We use a high-resolution Brillouin spectroscopy setup [30], [31] to characterize the high-frequency eigenmodes of the spheres. A 532 nm laser beam, focused into the sample, undergoes elastic scattering (Rayleigh scattering), and couples to the acoustic modulation of the material through an inelastic scattering process. The signal resulting from these processes is detected in backscattering configuration to resolve the mechanical eigenmodes of the system. Due to the sample conformation, the Rayleigh scattering is significantly larger than the inelastic scattering and determines a low-frequency region where the detection of Brillouin scattering is not possible, limiting our measurement range to higher frequencies ( > 3.5 GHz). In this measurement, the acoustic wave vector *q* ^2*αA*^ is related to the scattering angle (2*α*) as $q^{2\alpha A}=\frac{4\pi \sin\left(\alpha \right)}{\lambda_{0}}$ , *α* being the angle of incidence and *λ*_0_ the laser wavelength. Here, the reflecting substrate acts as a virtual light source, introducing the 2*αA* scattering geometry that is confined in the sample/substrate plane [32]. Even though the thickness of the polystyrene sphere layer is smaller than the laser beam focus, this scattering geometry still allows the observation of the nanosphere eigenmodes. To avoid inelastic coupling to surface acoustic modes of the substrate, the incident laser beam is polarized perpendicular to the scattering plane (s-polarization). The experimental measurement of the resonant modes for the isolated spheres is shown in Figure 2(a). The green shaded area represents the Rayleigh scattering signal, where Brillouin scattering is saturated, while the grey area represents our visible region. The Stokes and anti-Stokes contributions corresponds to the negative and positive frequencies represented by blue shaded peaks. By using oxygen plasma treatment to etch the sample, we were able to selectively reveal the resonant modes of the polystyrene spheres, effectively preventing any collective vibration. For the isolated spheres, a prominent peak is found around 5.8 GHz and less intense peaks are found around 6.7 GHz, 8.8 GHz and 11.9 GHz. It is important to underline the relationship between the size of the sphere and the frequency of the vibrational eigenmode: as the size of the spheres decrease, the frequency (energy) associated with their modes increases. As expected, touching and overlapping spheres, that did not undergo the reducing etching treatment, exhibit bands at lower energies due to their coupling as opposed to the isolated ones. Figure 2(c) is constructed using the peaks extracted from the spectra obtained for various incident angles on the samples for the Brillouin (blue, yellow, and green dots representing isolated, touching, and overlapping spheres, respectively). The 6 GHz band observed in the touching sphere system exhibits a slight shift to higher frequencies in the case of overlapping spheres. This finding is consistent with our computational results, which predict a band splitting; however, due to the limited spectral resolution of our experimental setup, we are unable to directly resolve this splitting. Experimental data are plotted along the calculated bands to have a clear vision of the different systems. The experimental data cannot be accurately represented in the reciprocal space because the in-plane orientation of the samples within the measured region is unknown. This uncertainty arises due to the fabrication technique and the small particle size, that requires near UV frequencies to allow diffraction patterns that are used to determine the orientation in larger sphere crystals [33], [34]. Therefore, although we cannot map the entire band diagram, we can estimate the energy levels, providing valuable insights into the system.

To complement the Brillouin spectroscopy, resolve the low-frequency vibrations of our systems obscured due to the Rayleigh elastic scattering, and probe the transport character of the collective modes, we use a pump-probe experimental technique [35]. For the pump-probe measurements, an intense femtosecond pulse (4 mW) is sent to the sample, causing it to expand through thermoelasticity and generate a strain pulse [36]. This pulse propagates through the sample, altering the refractive index caused by photoelastic interactions and creating a surface displacement of the spheres [37]. A delayed pulse, the so-called probe, then measures these changes by detecting the modulation in optical reflectivity. In our initial exploration, we employ local pump-probe experiments, where measurements are taken at the pumped location. Figure 2(b) shows the experimental data from this technique. At *t* = 0, the probe pulse initiates the measurement of the sample’s state in synchrony with its excitation by the pump pulse. An exponential decay is observed due to the thermal cooling of the sample. Additionally, oscillations emerge owing to the vibrations of the spheres. Fast Fourier transform (FFT) is used to determine the frequency of these oscillations once the exponential decay is subtracted as shown in the inset. The resonances also present a decay related to the losses of the system which are clearly higher on the overlapping spheres, related to the fact that attenuation is larger for higher frequencies [38]. Subsequently, various spots across samples were measured to confirm their homogeneity. Figure 2(c) depicts the normal angle measurements for the pump-probe (yellow and green lines).

When the spheres are isolated, the pump-probe technique does not show any vibrational mode in the low-frequency range of the spectrum as depicted in Figure 2(c). This absence of individual or collective vibrational states in the low energy region is in agreement with our numerical calculations and emphasizes the key role that sphere-to-sphere interactions play in driving the vibrational phenomena within the system. While measurements of high-frequency modes show minimal discrepancies between those touching and overlapping spheres systems, pump-probe measurements showed better resolution for the comparison of both of them. Touching spheres revealed a prominent peak at approximately 1.79 GHz. In contrast, overlapping spheres samples exhibited a distinct peak around 3.1 GHz, indicative of a subtle yet significant shift in the vibrational behavior induced by the sintering process. In addition, a shoulder at around 1.85 GHz observed in the overlapping spheres samples suggests the presence of a second band, as indicated by the calculations too. This observation aligns with the known effects of sintering, which involves the migration of material from the spheres to the necks, effectively closing the gap between adjacent spheres and altering the system’s vibrational dynamics in the process. If a further sintering process is carried out, a thin, unstructured, polystyrene film would form, resulting in a larger number of bands effectively closing the gap.

**Figure 3:**
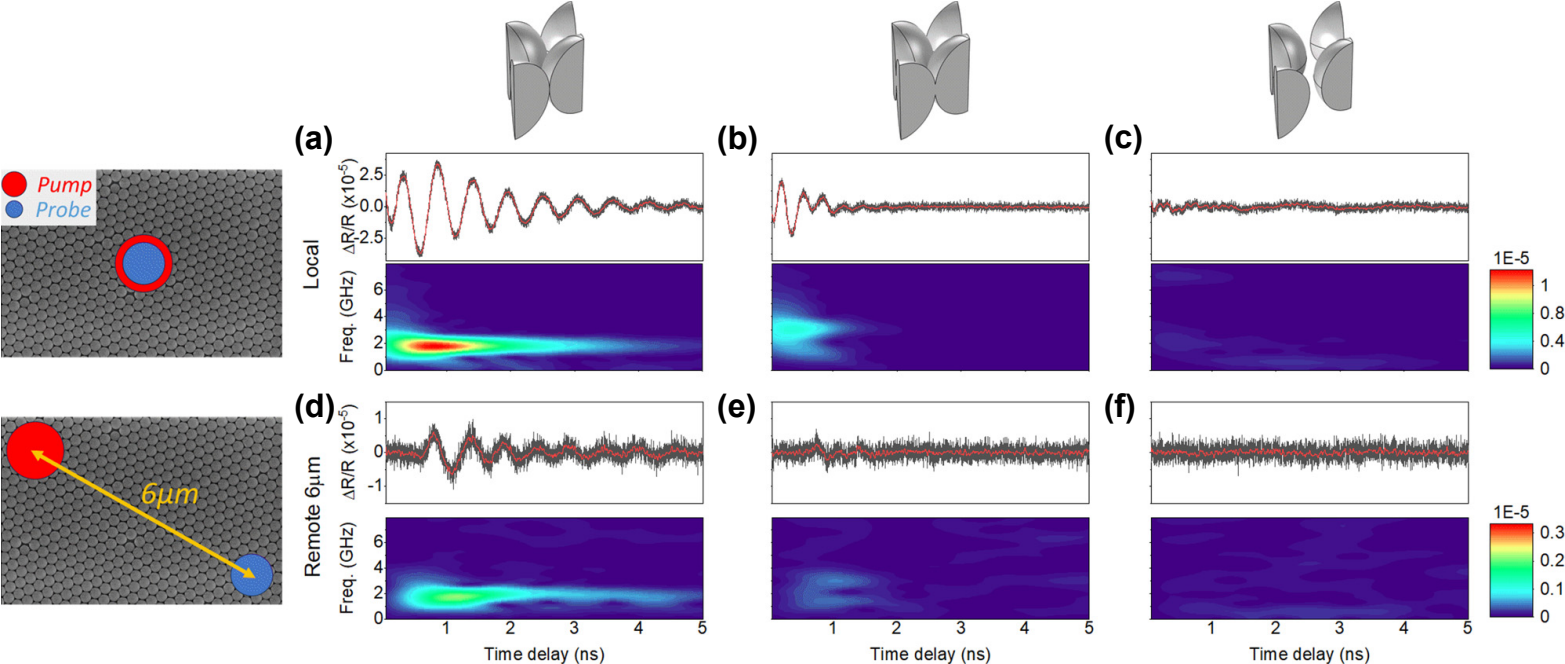
Pump-probe measurements for the three different systems. Figures (a), (b) and (c) show measurements with the pump and probe at the same position, while figures (d), (e) and (f) display measurements where the pump and probe are 6 μm apart. Signals are detected for both touching and overlapping spheres but not for the isolated ones, depicting a delay in the pulse for the non-local measurement. Black line – transient reflectivity signal after thermal background subtraction; red line – low-pass filtered signal; color map – short-time Fourier transform analysis.

We also explore the acoustic transport properties of vibrational modes in our structures using the pump-probe technique. For that, we displace the probe position with respect to the pump spot in a non-local configuration. Exploiting this approach, measurements were taken at varying distances from the pump position, allowing us to probe the propagation dynamics across different regions of the sample. Figure 3 presents the experimental data obtained for a local measurement compared with a measurement where the distance between the pump and probe is 6 μm. Measurements at various lengths (Figure S4 in Supplementary Information) revealed that the excitation pulse arrived at different times, providing evidence of energy transport along the samples. In these measurements, we do observe a clear signal in the touching spheres corresponding to a mode with a frequency of 1.80 GHz. In the overlapping spheres, while there are still some propagation, the signal propagates with significantly higher attenuation compared to the touching spheres. On the contrary, we do not observe such transport in the isolated spheres. Our interpretation for the isolated spheres system is that the absence of contact between spheres prevents the formation of collective modes and prevents transport. Meanwhile, in the overlapping spheres system, the melted contact points enable transport which is subject to dissipation owing to the viscoelasticity of the material. This observation again supports our numerical predictions and highlights the crucial role of sphere-to-sphere interactions in enabling collective vibrational modes in the system.

The speed of sound in polystyrene is approximately 2,400 m/s [39]. However, from this non-local characterization, our estimation using the results shown in Figure 3 suggests a higher velocity around 8,300 m/s for the touching spheres. This estimation has been done considering the time required for the wave packet center to travel between the 3 μm and the 6 μm positions (Figure S4(d) and (g) in the Supplementary Information). For the overlapping spheres, the estimation with the center of the packet with those same positions is around 6,700 m/s. When the system is placed on top of a substrate, new bands appear in the dispersion diagram, and the substrate thickness significantly influences the overall band structure, as shown in Figure S3. For the thickest substrate considered, only a few bands emerge compared to the free-standing system. The slope of one of these bands accounts for the high propagation speed observed, being its calculated value approximately 7,600 m/s. This wave is distinct from a standard surface acoustic wave, where energy transport is typically confined to a depth of about one wavelength in the substrate. Instead, it strongly reflects the interaction between the spheres and the substrate, with significant participation of the substrate in the transport of energy [40]. This is evident in Figure S2 of the Supplementary Information, where the surface strain distribution reveals a substantial contribution from the substrate to the overall energy transport. This insight opens new possibilities for application-oriented designs, where key parameters such as the substrate properties, sphere size and material, and contact conditions are all relevant and accessible parameters.

## 3 Conclusions

In summary, our study, combining Brillouin spectroscopy and pump-probe spectroscopy, has offered valuable insights into the system’s vibrational dynamics, clarifying the complex relationship between structure, composition, and vibrational behavior. The complete phononic band gap opens on a free-standing polystyrene monolayer for which the thermal annealing shown here is a feasible and easy solution for structural stability. The differences between isolated, touching and overlapping spheres are also shown along with the corresponding bandgaps. Experimentally, Brillouin spectroscopy allows to record high frequency modes for different wavevectors while the pump-probe technique gives information for the region where the Brillouin is obscured by Rayleigh scattering. Furthermore, the latter setup provides direct transport information. On one hand, the isolated spheres showed an absence of propagation. Conversely, signals were prominently detected in both the touching and overlapping sphere systems, indicating consistent wave propagation dynamics in these interconnected configurations. Moreover, overlapping spheres showed higher losses due to the larger amount of material on the necks of the system. These findings not only enhance our understanding of the system’s complexities but also open the door to further advancements and applications in vibrational spectroscopy and materials science.

## 4 Methods

*Sample fabrication*: Following the liquid–air interface technique, samples were prepared on Silicon wafers. The 202 nm monodisperse polystyrene microparticles were acquired from microparticles GmbH. Before use, the colloidal suspension was further diluted to a concentration of 1.67 % w/v. using ethanol-water (2:1 v/v). Sodium dodecyl sulfate was added to the water phase at a concentration of 0.1 mmol/L before colloidal addition. Particles were added with a micropipette on an approximately 45° tilted hydrophilic glass substrate over which they slid towards the water surface and collected by scooping the supernatant structure that formed with a silicon wafer substrate. To render the silicon wafer substrate hydrophilic, an oxygen plasma treatment was applied. Once the monolayer was collected, it was left drying on a 45° position. Samples with overlapping spheres were obtained by a thermal treatment, subjecting the samples to 105 °C for 10 min. Samples with isolated spheres were obtained by an oxygen plasma treatment using a low-pressure plasma system (Tetra, Diener electronic GmbH, Germany), under an applied power, pressure and duration of, respectively, 240 W (at 40 kHz), 0.3 Torr and 30 s.

*Experimental characterization*: High-resolution Brillouin spectroscopy was used with a 532 nm diode-pump solid-state laser (DPSS Excelsior-SpectraPhysics) spectra as light source and a Sandercock 3 + 3 Pass Fabry-Pérot interferometer spectrometer [41]. A lens with a 120 mm focal length and a 25 mm aperture was used to focus the beam onto the sample, resulting in a scattering area with an approximate diameter of 80 μm. We use the standard pump-probe technique in reflection geometry to study the low-frequency acoustic modes of the sample. A mode-locked Ti-sapphire laser with a central wavelength of 840 nm, pulse width of ∼140 fs, and 80 MHz repetition rate, serves as the light source. The laser is split into pump and probe beams using a polarizing beam splitter. The pump beam is modulated at 800 kHz with an acousto-optic modulator, and the probe beam passes through a mechanical delay line to introduce a controlled delay. In the local configuration, where the pump and probe spots overlap on the sample, the modulated pump and delayed probe beams are combined collinearly and directed normally to the 50× objective lens. Both beams are focused onto the sample with a ∼2μ m spot diameter. The average laser powers are 4 mW for the pump and 1.5 mW for the probe. The reflected probe beam is collected by a photodetector, and the output is fed to a lock-in amplifier, which synchronously records the in-phase component of the signal at the pump modulation frequency. Polarization filters are used to block any reflected pump beam from reaching the detector. In the non-local configuration, the position of the pump spot relative to the probe spot on the sample is controlled using a 4f lens setup. Moving the first lens adjusts the angle at which the pump beam enters the microscope objective while keeping it centered at the entrance pupil. This causes a lateral shift of the pump spot on the sample.

## Supplementary Material

Supplementary Material Details
