# Supplementary material for: A self-assembled two-dimensional hypersonic phononic insulator
Source: Nanophotonics. 2025 Oct 15;14(22):3569–77. doi: 10.1515/nanoph-2025-0141 (PMC12592634; doi:10.1515/nanoph-2025-0141)
Supplement: Supplementary file 1 — Supplementary Material Details [file j_nanoph-2025-0141_suppl_001.pdf]

Supplementary information for
A self-assembled two-dimensional hypersonic phononic insulator

Pedro Moronta,[1,2] Sathyan Sandeep,[3] Edson R. Cardozo de Oliveira,[3] Rafael J. Jiménez-Riobóo,[1] Norberto Daniel Lanzillotti-Kimura,[3,*] P. D. García,[1,†] and Cefe López[1]

[1]Instituto de Ciencia de Materiales de Madrid, (ICMM) Calle Sor Juana Inés de la Cruz 3, 28049 Madrid, Spain. Consejo Superior de Investigaciones Científicas(CSIC)

[2]Escuela de Doctorado, Universidad Autónoma de Madrid

[3]Université Paris-Saclay, CNRS, Centre de Nanosciences et de Nanotechnologies, 10 Boulevard Thomas Gobert, 91120 Palaiseau, France

S1. IMPACT OF THE SUBSTRATE ON THE PHONONIC INSULATOR PERFORMANCE

The phononic insulator is fabricated using polystyrene spheres with a diameter of 202 nm. However, its properties are influenced by several variables. Ideally, a free-standing monolayer would exhibit a perfect band gap in the GHz range, as shown in the dispersion relation presented in this manuscript. Due to manufacturing constraints, a substrate is required. Figure S1 illustrates a hybrid mode between the substrate and the spheres when the membrane thickness is one-sixth of the sphere diameter. As shown in Figure 1g of the main manuscript, these hybrid modes close the full band gap of the structure. To prevent this effect, the substrate's vibrations must be decoupled from the mechanical modes of the structure. Although a thicker substrate reduces this hybridization, it also introduces energy losses as a trade-off. A clear solution to both issues is to fabricate self-standing microspheres layers, a challenge we are currently working on.

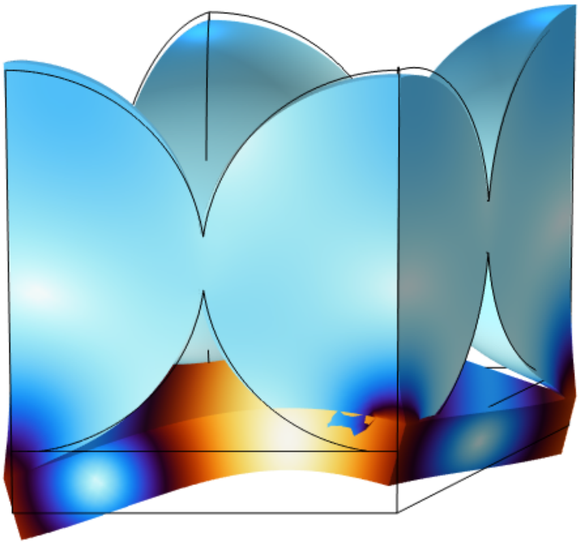

Supplementary Figure S1: Finite element solution for Γ-K direction at 3.5 GHz of a 202 nm diameter spheres monolayer on top of a 34 nm silicon membrane depicting a hybrid monolayer-substrate mode.

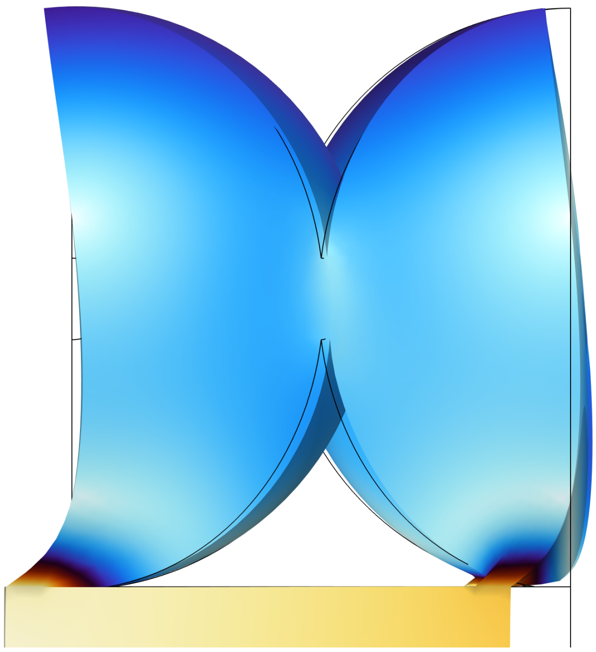

Supplementary Figure S2: Finite element solution for Γ-K direction at 4 GHz of a 202 nm diameter spheres monolayer on top of a thin silicon membrane depicting a hybrid monolayer-substrate mode.

It is important to note that the first hybridization occurs in the vertical direction. However, another form of hybridization arises in a monolayer on a substrate due to the surface-skimming longitudinal wave. This wave propagates even in thick substrates, reducing the complete bandgap to a partial bandgap. As illustrated in Figure S2, lateral displacement within the substrate exerts force at the spheres' contact points.

To understand how the substrate thickness affects the mechanical properties of the system, we analyze different configurations with an increasing substrate thickness. Figure S3 shows the dispersion relation for the overlapping-sphere system on substrates of varying thickness, ranging from a thin membrane relative to the sphere diameter to a substrate with the same thickness as the spheres. Although all these cases can be considered within the thin-substrate regime, they allow us to visualize the hybridization effect: hybridization between the spheres and the monolayer decreases as substrate thickness increases. The thinnest membrane heavily distorts the band dispersion across all energies. In the second system, hybridization occurs at higher energies in the $K - M$ direction, while it remains present in the $\Gamma - K$ direction. When the substrate thickness becomes comparable to the sphere diameter, the $\Gamma - K$ direction exhibits straight lines due to the small hybridization. These lines correspond to the modes shown in Figure S2.

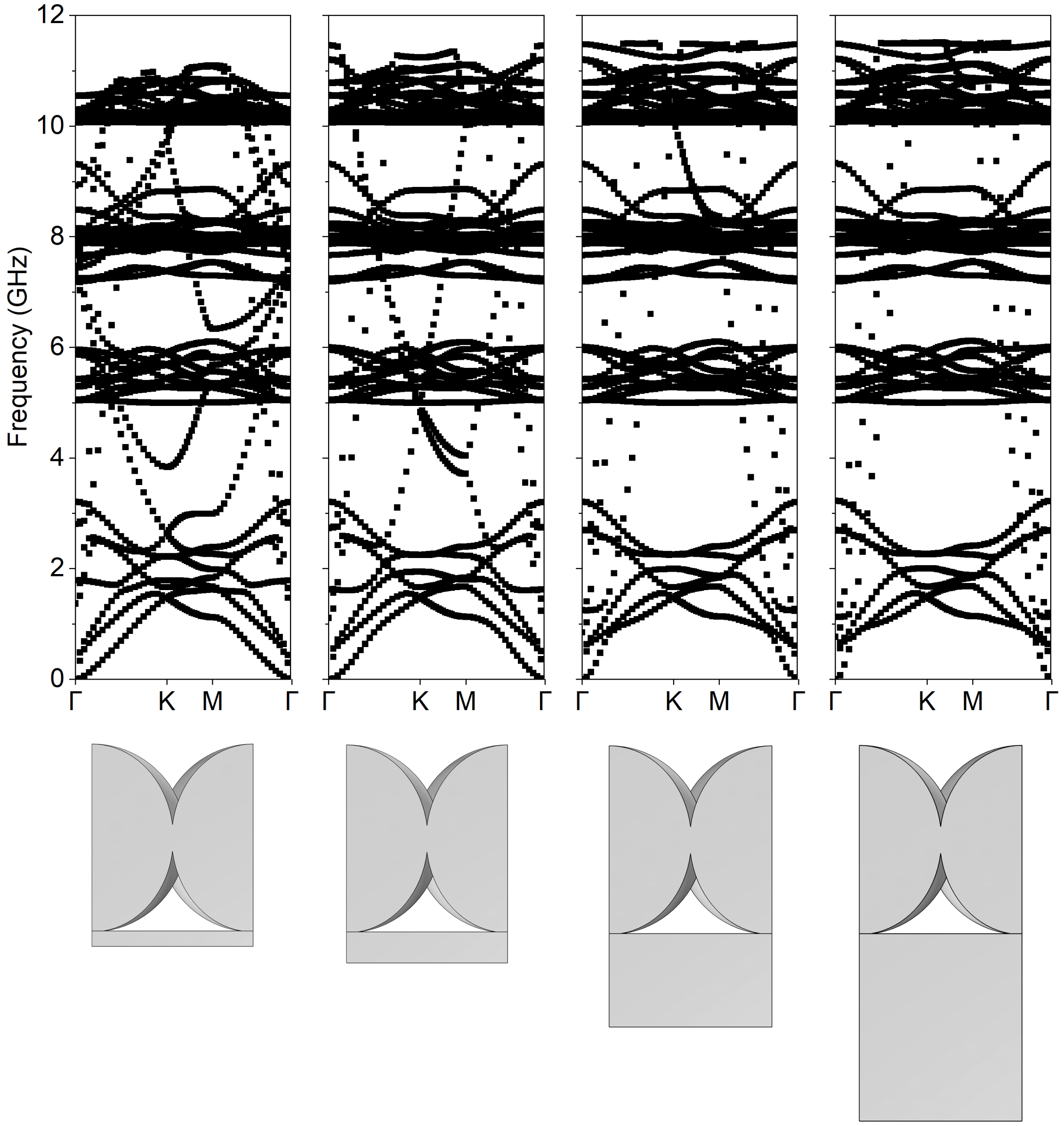

Supplementary Figure S3: Finite element solution for of a 202 nm diameter spheres monolayer on top of a silicon membrane with thickness **a)** 17 nm, **b)** 34 nm, **c)** 101 nm and **d)** 202nm.

S2. ACUSTIC-PHONON PROPAGATION.

To study the effect of acoustic-phonon propagation in our systems, we performed a non-local pump-probe characterization. Measurements were conducted on three fabricated systems—touching, overlapping, and separated spheres—at varying distances between the pump and probe. The experimental results are shown in Figure S4.

For the touching system, a well-defined signal is detected over a range of 0 µm to 9 µm, as shown in subplots *a, d, g*, and *j*. The signal arrives at different times and exhibits attenuation. In the overlapping sphere system (subplots *b, e, h*, and *k*), a weaker signal is observed due to greater attenuation at higher frequencies. Nonetheless, the signal remains detectable across various distances. For the non-touching sphere system, traces of a 7 GHz signal are observed in the local configuration, but no signal is detected in the

non-local measurements.

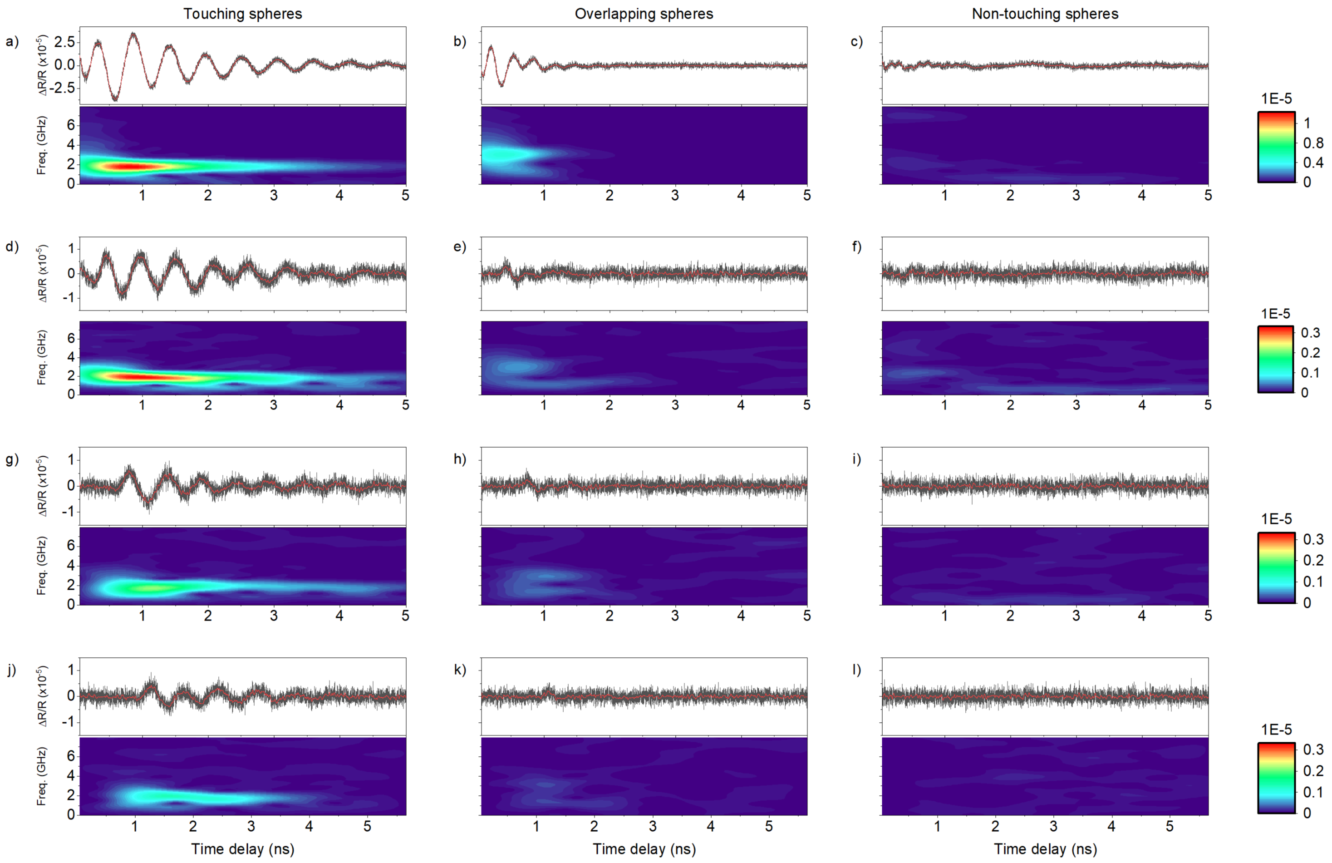

Supplementary Figure S4: Pump and probe experiments for the three systems under study of sphere monolayers on top of a silicon substrate *a), b), c)* for local measurements; *d), e), f)* for the pump 3 µm apart from the probe; *g), h), i)* for the pump 6 µm apart from the probe and *j), k) and l)* for the pump 9 µm apart from the probe.

As discussed in the manuscript, the calculated speed between the 3 µm and 6 µm measurements exceeds the expected value for the polystyrene system. To explain this high speed, substrate hybridization must be considered. Figure S2 illustrates this phenomenon, showing that it occurs even in thick substrates. The slope of the dispersion band associated with this hybridization corresponds to a group velocity of $v_g = 7600$ m/s, which aligns well with the speeds observed in this experiment.

* Electronic address: daniel.kimura@c2n.upsaclay.fr

† Electronic address: pd.garcia@csic.es
